# Overexpression of C16orf74 is involved in aggressive pancreatic cancers

**DOI:** 10.18632/oncotarget.10912

**Published:** 2016-07-28

**Authors:** Toru Nakamura, Toyomasa Katagiri, Shoki Sato, Toshihiro Kushibiki, Koji Hontani, Takahiro Tsuchikawa, Satoshi Hirano, Yusuke Nakamura

**Affiliations:** ^1^ Laboratory of Molecular Medicine, Human Genome Center, Institute of Medical Science, The University of Tokyo, Shirokanedai, Minato-ku, Tokyo, Japan; ^2^ Department of Gastroenterological Surgery II, Hokkaido University Graduate School of Medicine, Sapporo, Japan; ^3^ Division of Genome Medicine, Institute for Genome Research, The University of Tokushima, Kuramoto-cho, Tokushima, Japan; ^4^ Department of Medicine and Surgery, The University of Chicago, Chicago, Illinois, USA

**Keywords:** pancreatic cancer, novel gene, molecular target

## Abstract

Clinical outcome of pancreatic ductal adenocarcinoma (PDAC) has not been improved in the last three decades due to the lack of effective molecular-targeted drugs. To identify a novel therapeutic target for PDAC, we have performed genome-wide anamysis and found that *Homo sapiens* *chromosome 16 open reading frame 74 (C16orf74)* was up-regulated in the vast majority of PDAC. Overexpression of C16orf74protein detected by immunohistochemical analysis was an independent prognostic factor for patients with PDAC. The knockdown of endogenous *C16orf74* expression in the PDAC cell lines KLM-1 and PK-59 by vector-based small hairpin-RNA (shRNA) drastically attenuated the growth of those cells, whereas ectopic *C16orf74* overexpression in HEK293T and NIH3T3 cells promoted cell growth and invasion, respectively. More importantly, the endogenous threonine 44 (T44)-phosphorylated form of C16orf74 interacted with the protein phosphatase 3 catalytic subunit alpha (PPP3CA) via the PDIIIT sequence in the PPP3CA-binding motif within the middle portion of C16orf74 in PDAC cells. The overexpression of mutants of C16orf74 lacking the PDIIIT sequence or T44 phosphorylation resulted in the suppression of invasive activity compared with wild-type C16orf74, indicating that their interaction should be indispensable for PDAC cell invasion. These results suggest that C16orf74 plays an important role for PDAC invasion and proliferation, and is a promising target for a specific treatment for patients with PDAC.

## INTRODUCTION

Pancreatic ductal adenocarcinoma (PDAC) reveals worse prognosis than any other types of malignant tumor. The incidence of PDAC is increasing by 1.2% per year, and an estimated 53,070 new cases of pancreatic cancer were expected to occurred in the US in 2016 [1]. Only surgical resection offers a potential cure, but surgical resection is possible in only approximately 20% of patients with PDAC. The poor prognosis of this malignancy is probably due to two major reasons, the difficulty of early diagnosis and the generally poor response to current therapies, with a 5-year survival rate of 7% [1]. Systemic chemotherapy is the standard treatment for patients with metastatic PDAC; however, the median overall survival is only 11.1 months with FOLFIRINOX (a combination of oxaliplatin, irinotecan, fluorouracil, and leucovorin), 8.5 months with gemcitabine plus nab-paclitaxel, and 6.24 months with gemcitabine plus erlotinib (an epidermal growth factor receptor (EGFR) tyrosine kinase inhibitor) [2–4]. In combination chemotherapy, such as FOLFIRINOX, side effects including hematological toxicity, febrile neutropenia, sensory neuropathy, and gastrointestinal toxicity are higher than for single-agent gemcitabine; therefore, guidelines recommend that FOLFIRINOX be used in patients ≤75 years of age, with a good performance status of 0 or 1 and a level of bilirubin ≤1.5 ULN [5].

Molecular-targeted therapies, such as receptor tyrosine kinase inhibitors, have been developed for PDAC, and gemcitabine plus erlotinib therapy has been established. However, these drugs have failed to achieve significant improvement (only 2 weeks extension in median overall survival compared with single-agent gemcitabine) against PDAC [4]. Moreover, there is no evidence supporting the use of any other molecular-targeted therapies in PDAC, including cetuximab (a monoclonal antibody targeting epidermal growth factor receptor), bevacizumab (a monoclonal antibody targeting vascular endothelial growth factor A) or other inhibitors [6, 7].

Hence, the development of new effective therapies based on the identification of PDAC-specific molecular targets is urgently needed. We determined detailed expression profiles of PDACs using a genome-wide cDNA microarray containing approximately 27,000 genes, in combination with laser microdissection to obtain pure populations of cancer cells [8], and identified and characterized a novel gene termed *C16orf74* that is frequently over-expressed in pancreatic cancer specimens.

The *C16orf74* has been reported as *Homo sapiens* chromosome 16 open reading frame 74 (NM_206967.2) and is located on chromosome 16q24.1. This gene was shown to be associated with tumor necrosis factor (TNF)-alpha as well as hypoxic condition [9–11]. Moreover, several reports have indicated that *C16orf74* expression is a potential prognostic factor in several types of cancer [10, 12–15], but the pathophysiological functions of the *C16orf74* gene in PDAC cells have not been elucidated. In this report, we demonstrate that the *C16orf74* gene product interacts with the protein phosphatase 3 catalytic subunit alpha (PPP3CA) and is indispensable for invasion and proliferation of PDAC cells. Accordingly, we suggest that *C16orf74* is a potential therapeutic target for the development of anticancer drugs for the treatment of PDAC.

## RESULTS

### Identification of C16orf74 as an up-regulated gene in pancreatic cancer cells

**Figure 1:**
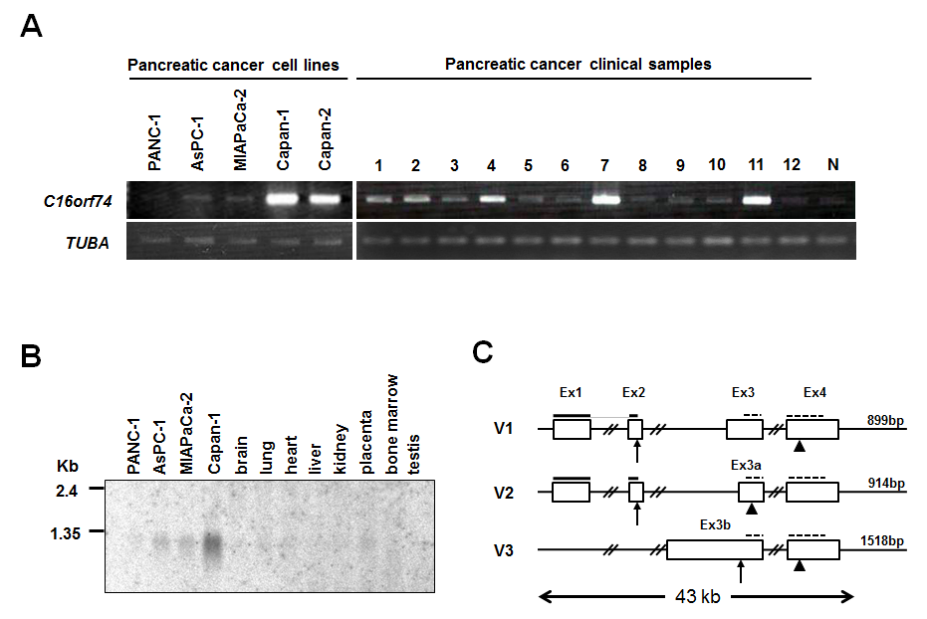
Up-regulated expression of *C16orf74* in pancreatic cancer cells and gene structure **A.** Semi-quantitative RT-PCR analysis in 5 pancreatic cancer cell lines and 12 clinical samples compared with a normal pancreas duct (N). The amount of RNA was normalized by *tubulin, alpha* (*TUBA*). The positions of *C16orf74* PCR primer are shown as dotted lines on Figure 1C. **B.** Northern blot analysis of the expression levels of *C16orf74*-V1/V2 transcripts in pancreatic cancer cell lines compared with normal tissues. **C.** Genomic structure of the three *C16orf74* variants (denoted V1, V2, and V3) spanning an approximately 43-kb region on 16q24, including four exons (Ex). Arrows and arrowheads indicate start and stop codons, respectively. The positions of V1/V2-specific and common probes for Northern blot analysis are shown as bold lines on V1, V2 and dotted lines on V1,V2,V3, respectively.

We verified by semi-quantitative RT-PCR that C16orf74 was up-regulated in 10 of 12 pancreatic cancer specimens compared with normal pancreatic ducts, and was up-regulated in capan-1, capan-2 pancreatic cancer cell lines compared with normal pancreatic ducts, although it was observed a weak band in normal duct cells. (Figure 1A). Subsequent northern blot analysis using a *C16orf74* cDNA fragment confirmed the overexpression of the approximately 1-kb transcript in Capan-1, Miapaca-2 and Aspc-1 cells. *C16orf74* was not expressed in normal human organs including the brain, lung, liver, kidney, placenta, bone marrow and testis (Figure 1B).

Because the EST sequence of the *C16orf74* gene in the National Center for Biotechnology Information (NCBI) database (Accession: BE875115; 586bp) is smaller than the approximately 1-kb transcript shown in Figure 1B, we screened the full-length cDNA clone from a cDNA library prepared from pancreatic cancer cell lines (see Materials and Methods) and isolated three different isoforms (Figure 1C). The three transcriptional variants were denoted *C16orf74*-V1 (GenBank Accession: AB115764), -V2 (AB115765) and -V3 (AB115766) and consist of 899, 914 and 1518 nucleotides that encode 76, 20 and 64 amino acids, respectively, as shown in Figure 1C. The amino acid sequences of these three variants were shown in Supplemental Figure 2.

The V2 variant has a unique exon 3 (exon 3a) that is 22-bp shorter at the 5’ end than exon 3 of V1, resulting in a frame shift within exon 3a and an early stop codon. The V3 variant lacks exons 1 and 2 and has a unique exon 3 (exon 3b) that is 834-bp longer at the 5’ end than exon 3 of V1. Although this variant has the first ATG within exon 3b as the initial codon, we did not confirm that the protein is translated from this transcript because the consensus Kozak sequence was not conserved around this ATG sequence. Northern blot analysis with multiple normal human tissues and PDAC cell lines revealed that the V1 and V2 transcripts but not V3 are up-regulated in PDAC cell lines compared with normal tissues (Figure 1B, Supplemental Figure 1A). Moreover, among 23 normal organs, the V1 and V2 transcripts were also highly expressed in the placenta and weakly in the thyroid (Supplemental Figure 1A, 1B), whereas expression of V3 was relatively high in the lymph node and very low levels of expression were observed in the stomach, trachea and bone marrow (Supplemental Figure 1A).

**Figure 2:**
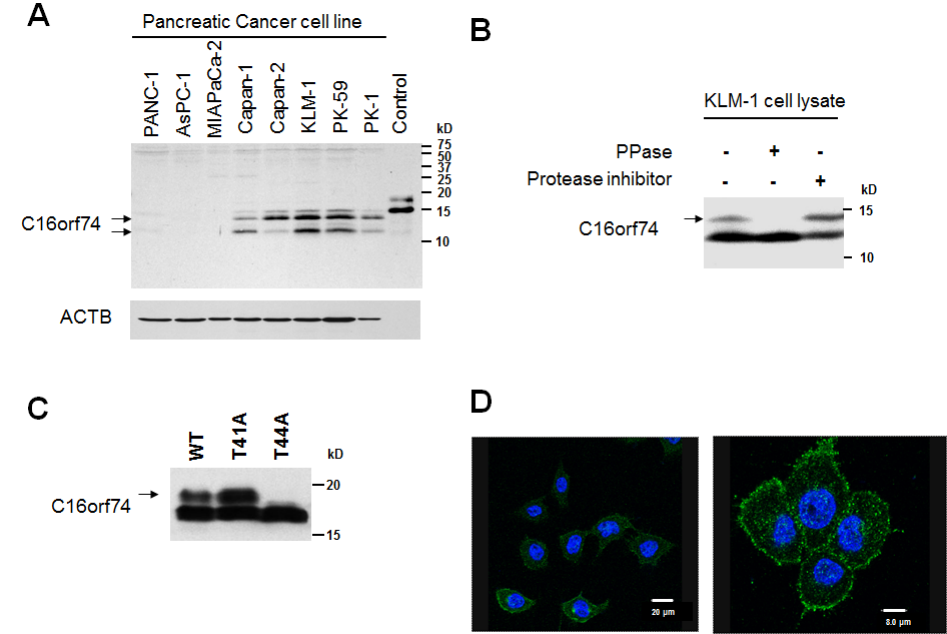
Protein expression, modification, and localization of C16orf74 in pancreatic cancer cells **A.** Western blot analysis of the expression levels of C16orf74 in pancreatic cancer cell lines. Control: Flag-tagged C16orf74-overexpressed diluted cell lysate. **B.** Phosphorylated form (arrow) of endogenous C16orf74 in KLM-1 cells, as examined by Western blot analysis using an anti-C16orf74 polyclonal antibody. The upper band disappeared when the cell lysate was incubated with lambda phosphatase (PPase (+)). **C.** Phosphorylation at threonine 44 (T44) of C16orf74. Flag-tagged wild type (WT), T41A and T44A mutants of C16orf74 were used to transfect COS-7 cells. The phosphorylated form of wild-type C16orf74 (arrow) was disappeared in the T44A mutant. **D.** Immunocytochemical analysis in a pancreatic cancer cell line (PK-1) using the anti-C16orf74 antibody, demonstrating the plasma membrane localization of endogenous C16orf74 (Green). DAPI staining is shown in blue.

To investigate endogenous C16orf74 expression at the protein level, we generated an anti-C16orf74 polyclonal antibody which can recognize V1 and V3 proteins, but not V2 protein, and performed Western blot analysis using extracts from pancreatic cancer cell lines as well as COS7 cells in which C16orf74-V1 protein expression vector was exogenously introduced. We detected the two major bands in COS7 cells without any nonspecific bands and pancreatic cancer cells (Figure 2A). A few minor additional bands were observed in pancreatic cancer cells, but we did not characterize further. These minor bands may correspond to another isoform(s) that we could not identify by cDNA cloning, to modified C16orf74-V1 proteins, or to different proteins that the antibody might have cross-reacted. Moreover, the results of western analysis that indicated high level of C16orf74-V1 protein in Capan-1, Capan-2, KLM-1, PK-59 and PK-1 cells, but not in Panc-1, Aspc-1 and Miapaca-2 cells (Figure 2A), are consistent with the quantitative RT-PCR (qRT-PCR) results. Hence, we focused on the V1 protein in subsequent functional analyses.

To investigate the possible post-translational modification of the C16orf74-V1protein, we performed a lambda phosphatase assay and observed a band corresponding to the phosphorylated form (arrow). This band disappeared when the cell extracts were incubated with lambda phosphatase, but not with protease inhibitor (Figure 2B). The Eukaryotic Linear Motif (ELM) prediction tool (http://elm.eu.org/index.html) indicated that the C16orf74-V1 protein possesses a predicted phosphorylation site at threonine 44 (T44) in its middle portion (Supplemental Figure 2). Substitution of T44 to alanine (T44A) abolished the phosphorylated band, whereas the T41A substitution did not, indicating that T44 is a major phosphorylation site of the C16orf74 protein (Figure 2C). Moreover, immunocytochemical analysis with an anti-C16orf74 antibody revealed that endogenous C16orf74 is localized under the plasma membrane (Figure 2D). However, although C16orf74 has no putative transmembrane domain, it is predicted to possess N-myristoylation at glycine 2 (G2) by *in silico* analysis (Supplemental Figure 2). Accordingly, we suspected that C16orf74 is anchored to the plasma membrane *via* N-myristoylation at G2, although further analysis of this modification of the C16orf74 protein is necessary.

To further investigate C16orf74 expression in PDAC surgical specimens and normal tissue sections, we performed immunohistochemical staining with an anti-C16orf74 antibody and observed strong staining in ductal cancer cells, whereas no staining was observed in the corresponding normal pancreatic ductal cells (Supplemental Figure 3A). Moreover, consistent with the results of the Northern blot analysis, no expression was observed in the kidney, liver, heart, and lung (Supplemental Figure 3B).

### Correlation between C16orf74 expression pattern and PDAC patient prognosis

**Figure 3:**
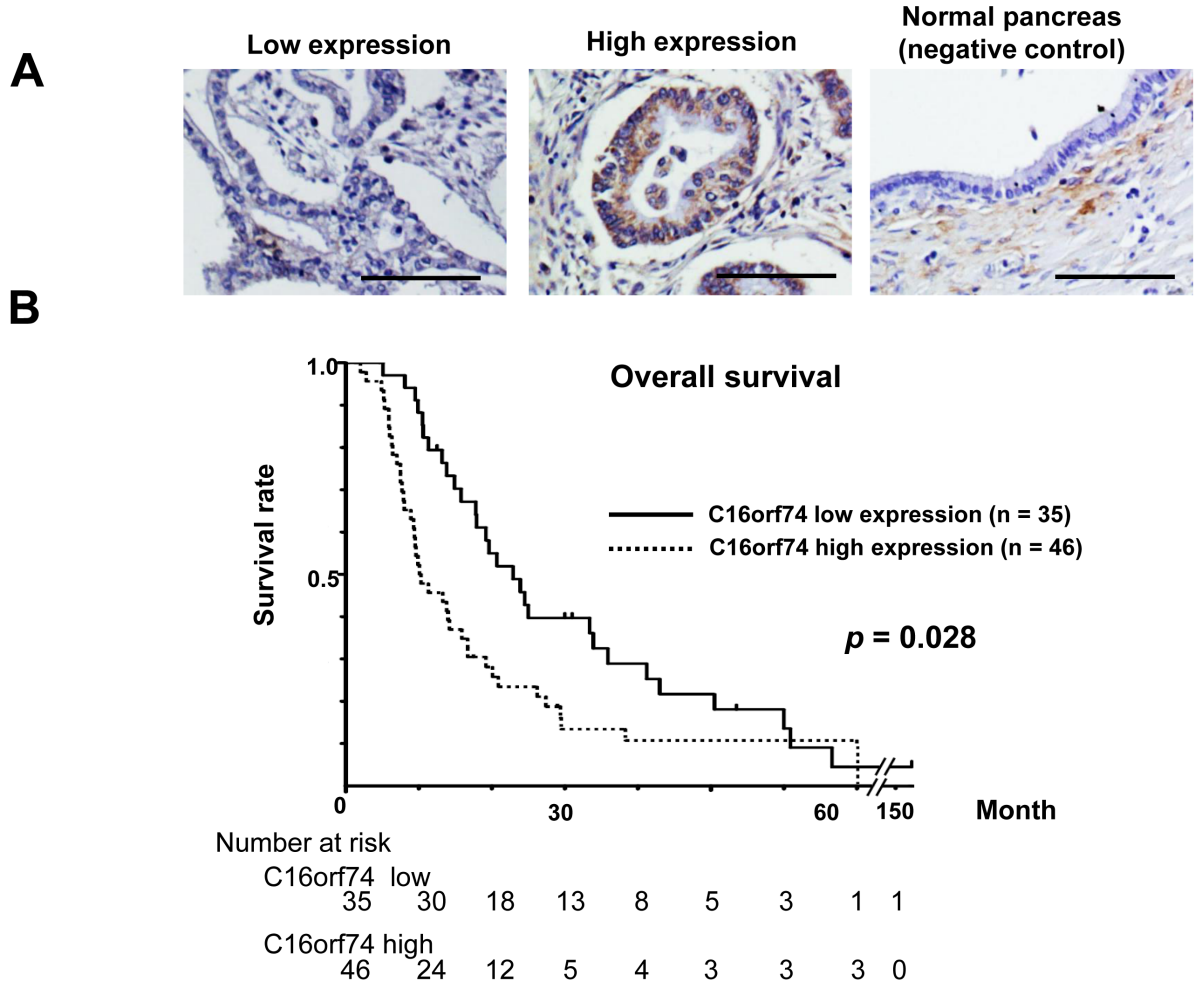
Expression of C16orf74 in human PDAC tissues and its correlation with overall survival **A.** Immunohistochemical staining of C16orf74 in human PDAC tissues and normal pancreatic tissues. Scale bar: 100 μm. **B.** Kaplan-Meier survival analysis of overall survival based on C16orf74 expression in 81 resected PDAC samples. The C16orf74-positive group exhibited significantly lower survival rates (*p* = 0.028).

To assess the clinicopathological significance of C16orf74 overexpression in PDAC, we conducted immunohistochemical staining of a tissue microarray from 81 PDAC cases that underwent curative surgical resection. The relationship between the overall survival and the expression level of C16orf74 was evaluated by the Kaplan-Meier Method (Figure 3). The C16orf74 high-expression group (with > 10% positive cancer cells in the tissue section) had significantly worse prognosis than the C16orf74 low-expression group (with ≤ 10% or no positive cancer cells in the tissue section) (median survival 10.1 months in the high-expression group *vs*. 20.7 months in the low-expression group, *p* = 0.028). The clinicopathological data and C16orf74 expression status are shown in Table 1. Multivariate analysis using a Cox proportional-hazard model indicated that lymph node metastasis status and the C16orf74 expression level were independent poor prognostic factors for patients with surgically-resected PDAC (2.61; 95%CI (1.51-4.53) and 2.05; 95%CI (1.25-3.36) at relative risk, respectively).

**Table 1:** Clinicopathological characteristics of pancreatic adenocarcinoma patients according to C16orf74 expression

		C16orf74	C16orf74	
Factor		Low expression	High expression	*p* value
		*n* = 35	*n* = 46	
Age; median (range)		67 (42-82)	65 (44-89)	0.74
Sex				
	Male	25	23	0.052
	Female	10	23	
Tumor size (mm);median (range)		29.5 (10.0-60.0)	35.0 (15.0-73.0)	0.034*
T stage				
	1-2	1	0	0.25
	3-4	34	46	
Differentiation				
	Well	6	6	0.55
	Moderate	17	19	
	Poor	12	18	
	Adenosquamous	0	2	
Tumor site				
	Ph	26	35	0.85
	Pb, Pt	9	11	
Lymph node metastasis				
	+	25	28	0.32
	-	10	18	
Lymphatic invasion				
	+	25	34	0.80
	-	10	12	
Venous invasion				
	+	31	40	0.76
	-	4	6	
Perineural invasion				
	+	32	43	0.73
	-	3	3	
UICC stage				
	- 2a	10	18	0.34
	2b	24	28	
	3 -	1	0	
CEA (ng/mL); median (range)		4.1 (1.3-34.4)	4.9 (1.1-70.8)	0.11
CA19-9 (U/mL); median (range)		127.8 (1.0-6606)	165.6 (1.0-19238)	0.22

Ph: pancreatic head, Pb: pancreatic body, Pt: pancreatic tail, UICC: Union for International Cancer Control, U/mL: Unit/mL, *p < 0.05.

### Effect of C16orf74 on cell growth and cell invasion

**Figure 4:**
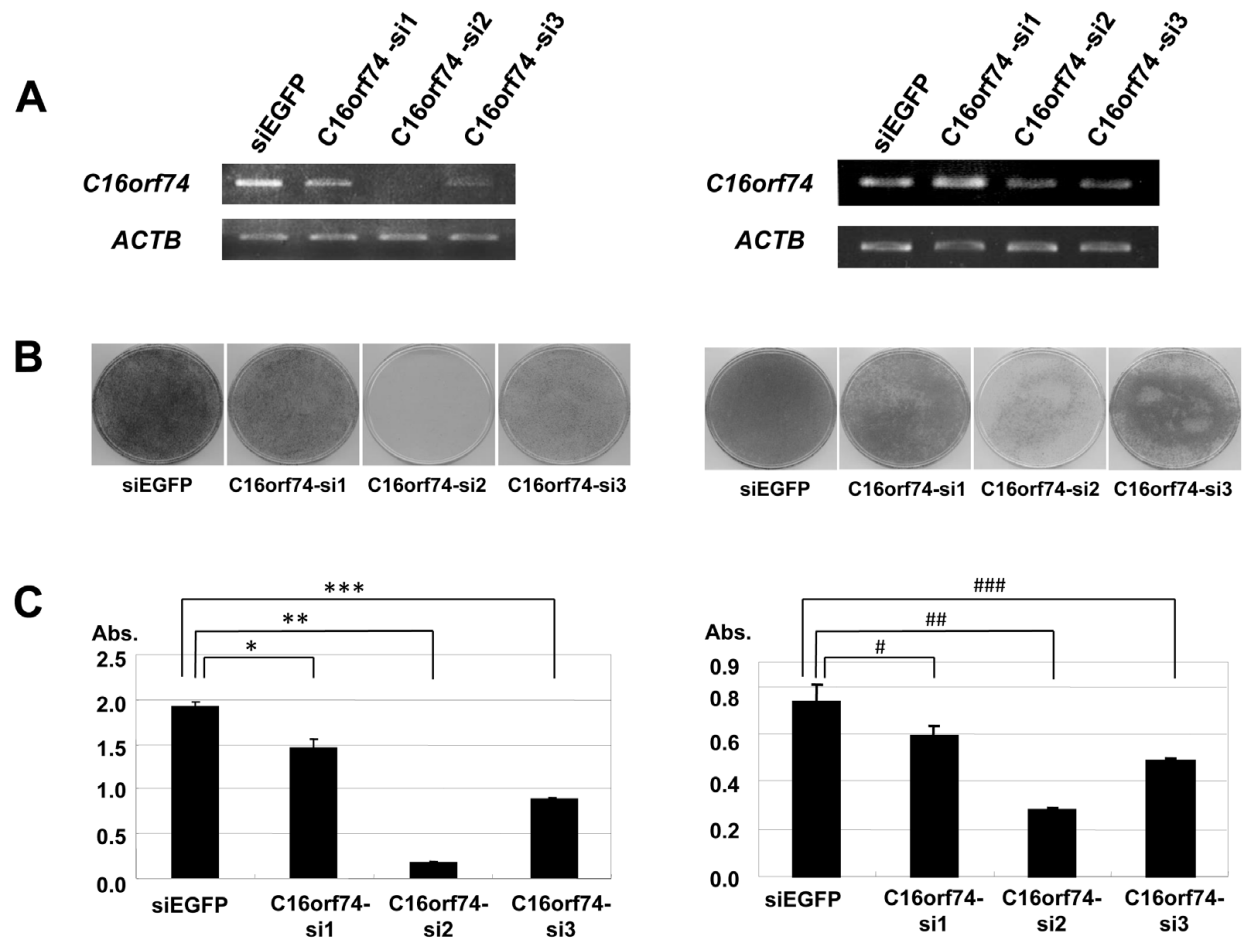

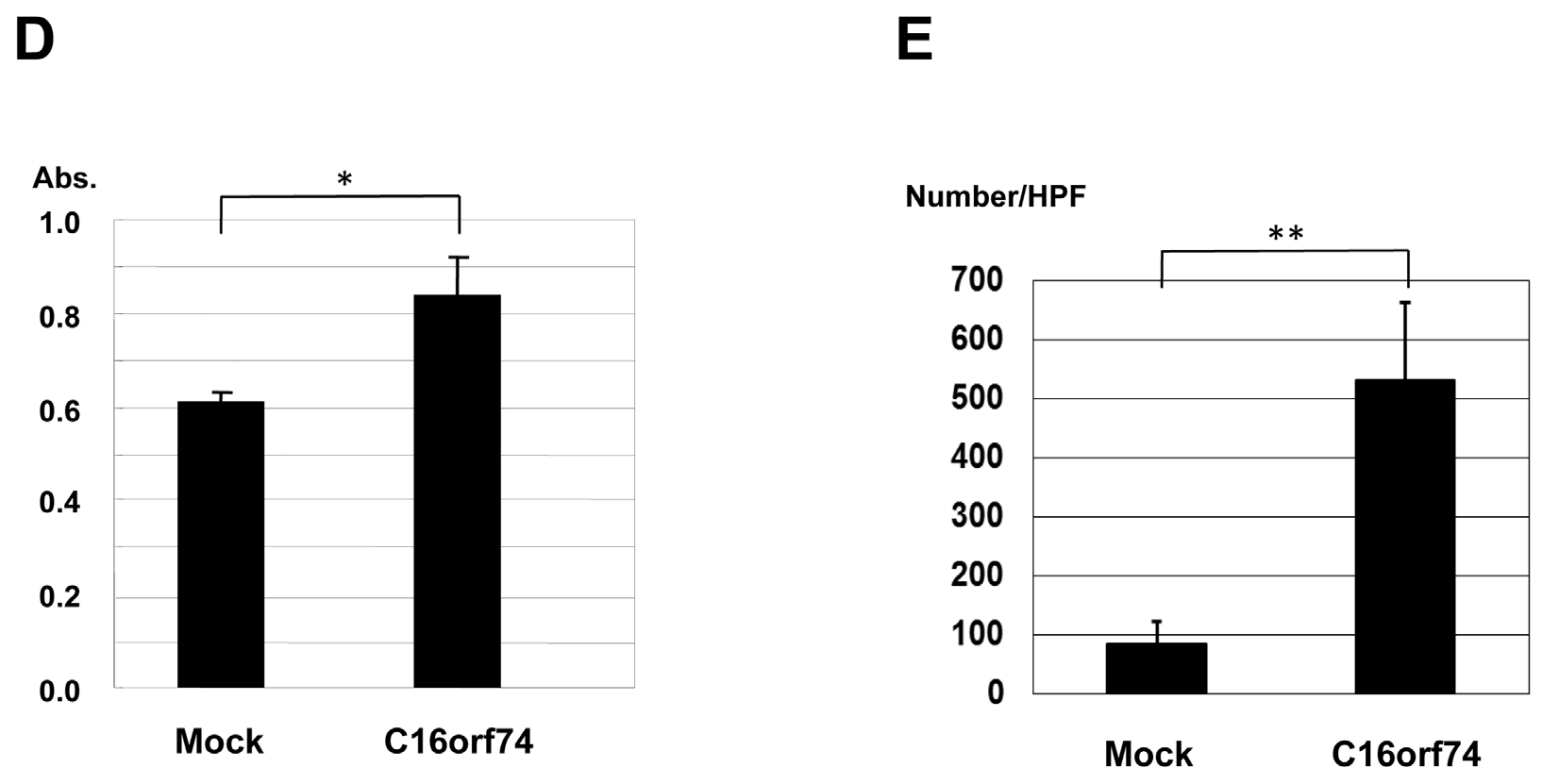
Effect of knockdown of C16orf74 on cancer cell growth Three *C16orf74* siRNA expression vectors (*C16orf74*-si1, -si2, and -si3) and an *EGFP* siRNA expression vector (si*EGFP*) as a negative control were transfected into KLM-1 cells (left) and PK-59 (right). **A.** The effect of knockdown on the *C16orf74* transcript was validated by RT-PCR with *ACTB* expression as a quantitative control. *C16orf74*-si2 displayed a strong knockdown effect, whereas the effect of *C16orf74*-si3 was modest. *C16orf74*-si1 and si*EGFP* failed to exhibit any effect on the level of the *C16orf74* transcript. **B.** Transfection with the *C16orf74*-si2 vector resulted in a drastic reduction of the number of colonies in the colony formation assay compared with cells transfected with si*EGFP* or *C16orf74* -si1. **C.** Transfection with the *C16orf74*-si2 vector resulted in a drastic reduction of the number of colonies in the MTT assay compared with cells transfected with si*EGFP* (control) or *C16orf74* -si1. The number of viable cells was consistent with the colony formation assay. For the KLM-1 cells (left), cell viability were 24% reduction in *C16orf74*-si1 (*** *P* < 0.0001), 91% reduction in *C16orf74*-si2 (** *P* < 0.0001), 54% reduction in *C16orf74*-si3 (*** *P* < 0.0001). For the PK-59 cells (right), cell viability were 34% reduction in *C16orf74*-si1 (# *P* < 0.0001), 62% reduction in *C16orf74*-si2 (## *P* < 0.0001), 39% reduction in *C16orf74*-si3 (### *P* < 0.0001). **D.** The *C16orf74* expression vector or Mock vector was transfected into HEK293T cells. The MTT assay revealed enhanced cell growth in C16orf74-over-expressing cells (*P* = 0.001). **E.** Effect of over-expression of C16orf74 on cell invasion. The *C16orf74* expression vector or Mock vector was transfected into NIH3T3 cells. The Matrigel invasion assay revealed enhanced cell invasion by C16orf74 over-expressing cells (*P* = 0.03).

To assess the biological roles of *C16orf74* in PDAC cell growth, we conducted loss-of-function studies. We examined the effect of knockdown of *C16orf74* expression by mammalian vector-based small hairpin RNA interference (shRNA) on the cell growth of KLM-1 and PK-59 cells by colony-formation and MTT assays. Semi-quantitative RT-PCR revealed a significant reduction of the amount of *C16orf74* transcript in both cells lines when transfected with the shRNA constructs (si#2 and si#3) but not those with the control shRNA (shEGFP) (Figure 4A). Consistent with the effect of *C16orf74* knockdown, MTT and colony formation assays clearly revealed significant growth inhibition of both cell lines (Figure 4B, 4C). To further confirm the growth-promoting effects of *C16orf74* overexpression, we conducted an MTT assay of HEK293T cells transiently transfected with *C16orf74*-expressing plasmids. *C16orf74*-overexpressing cells grew significantly faster than cells transfected with mock plasmid, suggesting a growth-promoting effect of C16orf74 (Figure 4D).

We also performed a Matrigel invasion assay to investigate the effect of C16orf74 overexpression on cellular invasion ability. The invasion of NIH3T3 cells transfected with C16orf74 was significantly enhanced compared with the control cells transfected with mock plasmid (Figure 4E), suggesting that C16orf74 contributes to the highly malignant phenotype of PDAC cells.

### C16orf74-PPP3CA interaction is indispensable for invasion

**Figure 5:**
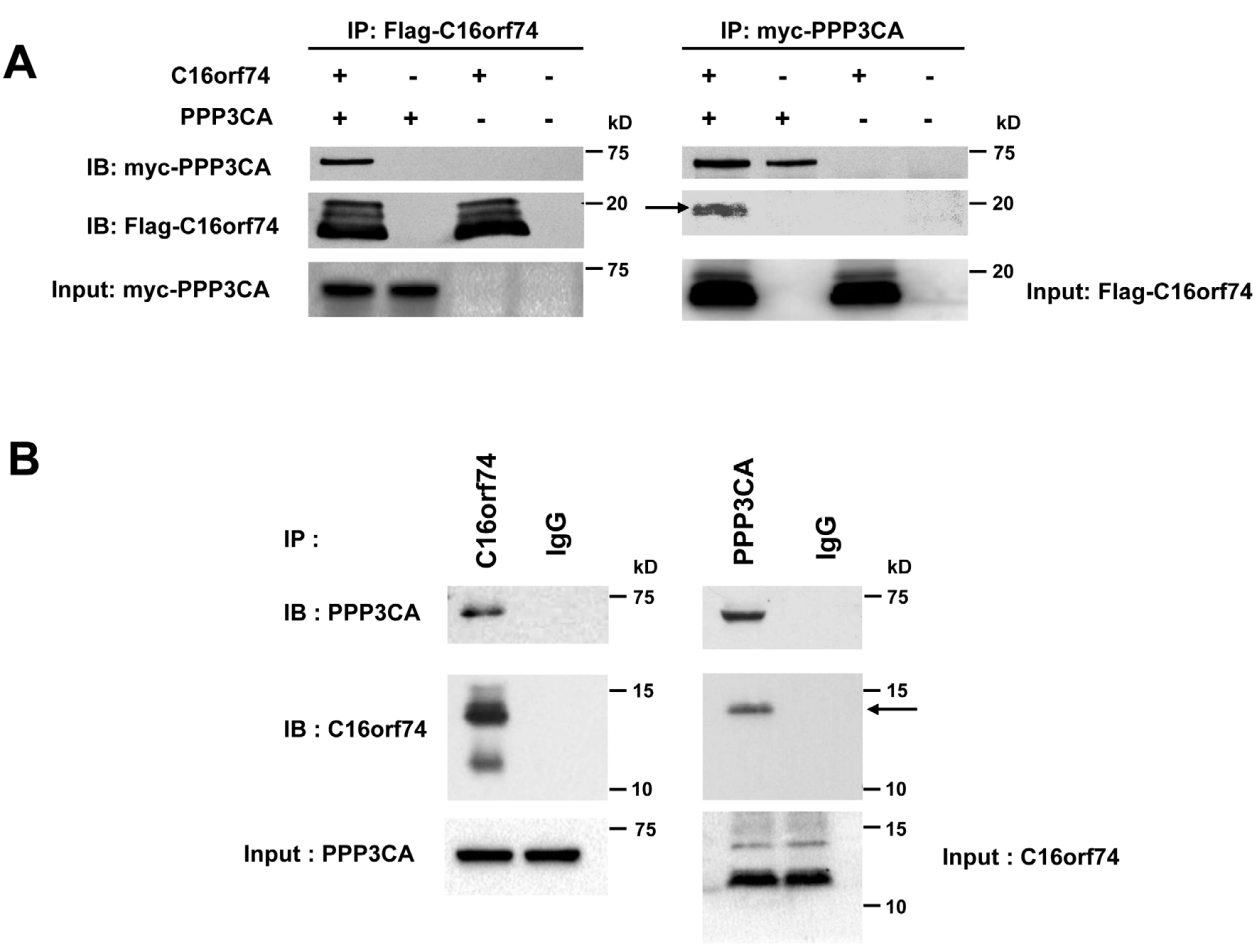

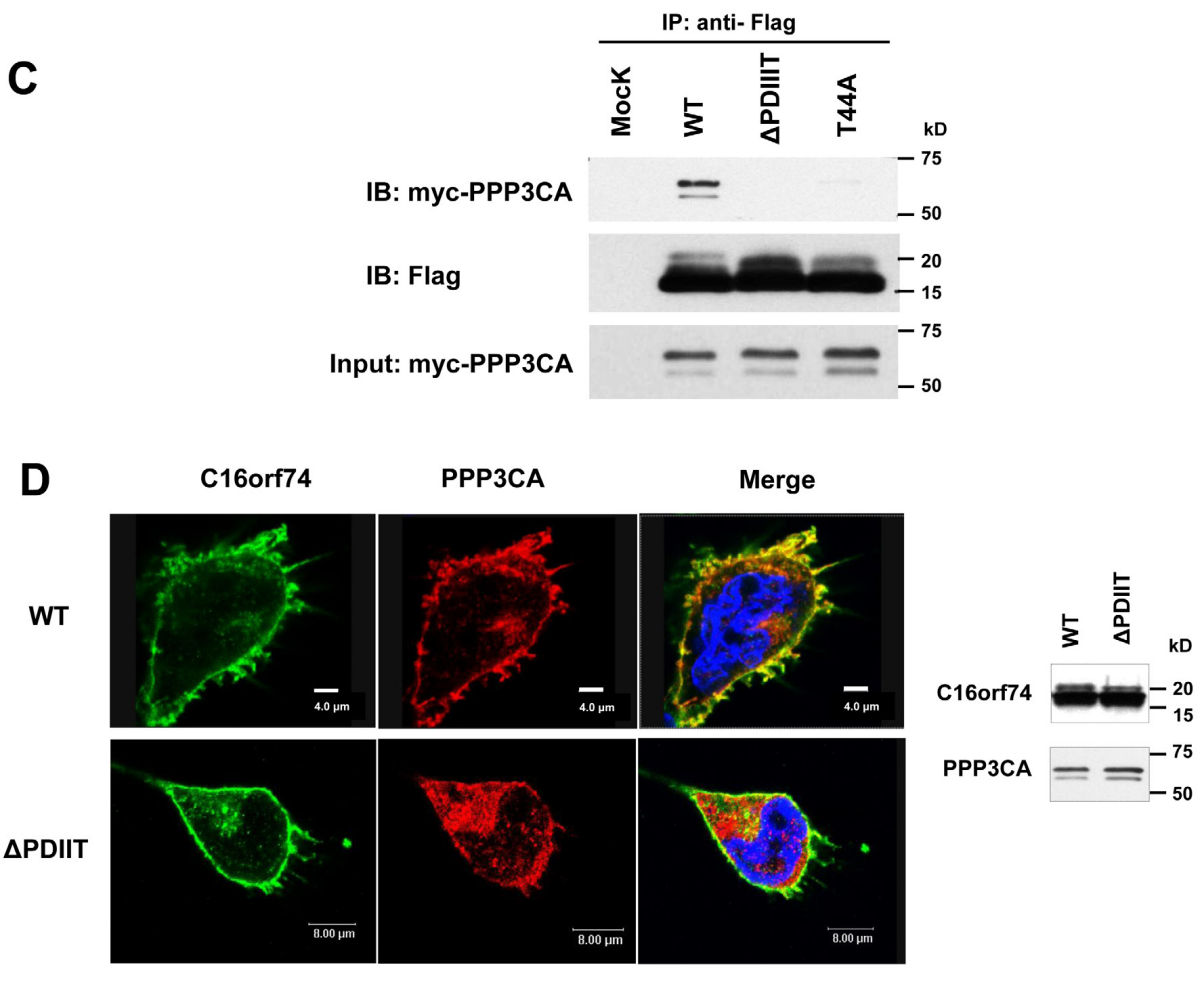

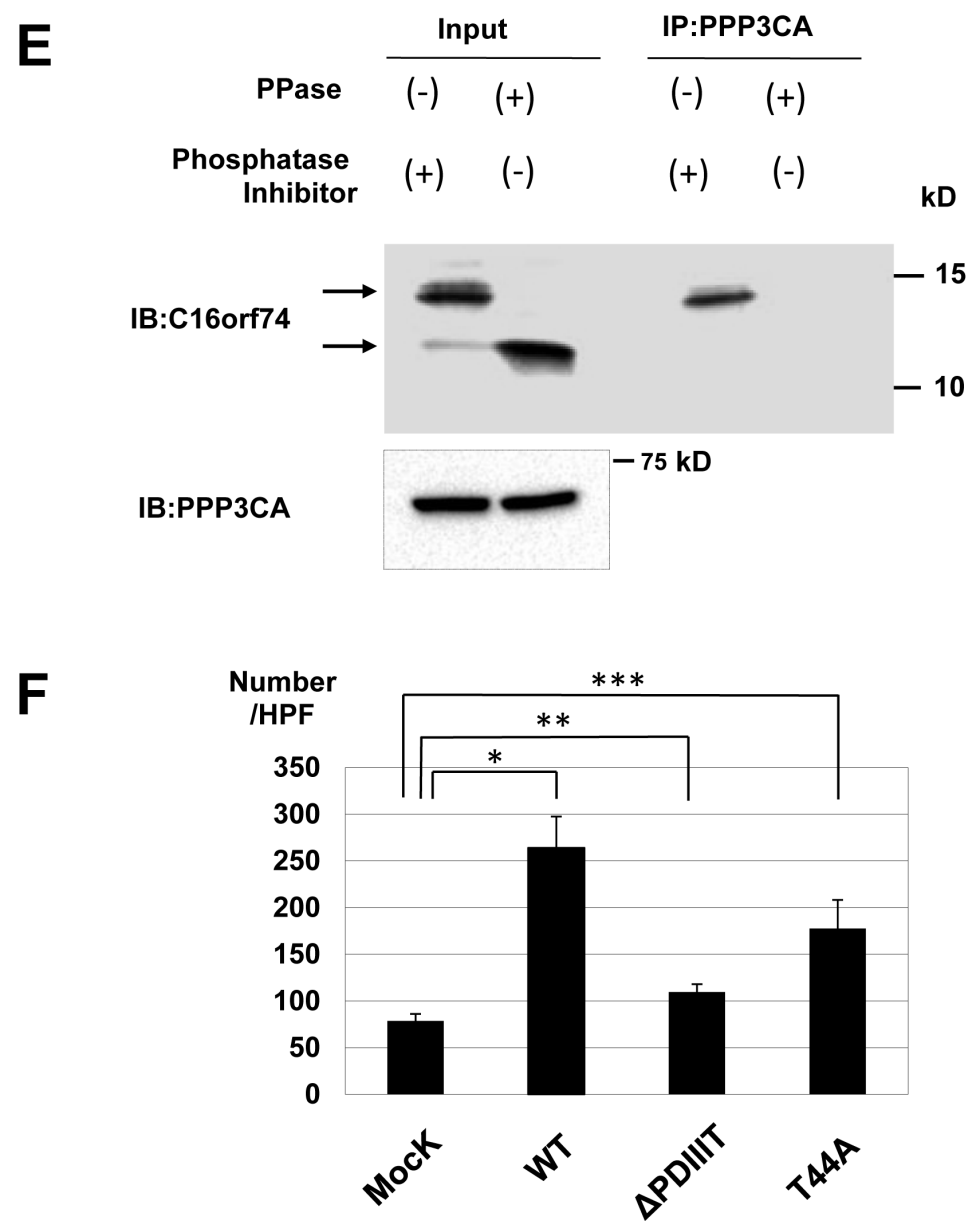
Interaction between C16orf74 and PPP3CA **A.** *In vitro* exogenous association of C16orf74 and PPP3CA. The Flag-tagged C16orf74 construct or vector alone was cotransfected with a myc-tagged PPP3CA construct into HEK293 cells. Cell lysates were immunoprecipitated using mouse anti-Flag antibody (left) or anti-myc antibody (right). Immunoblotting of the immunoprecipitates with rabbit anti-Flag or anti-myc antibodies revealed a specific interaction between the phosphorylated form of C16orf74 (arrow) and PPP3CA. **B.** *In vitro* endogenous association of C16orf74 and PPP3CA from Capan-1 pancreatic cancer cells, which endogenously express high levels of both C16orf74 and PPP3CA. Capan-1 cell lysates were immunoprecipitated using anti-C16orf74 antibody (left) or anti- PPP3CA antibody (right). Immunoblotting of the immunoprecipitates with anti-C16orf74 antibody or anti-PPP3CA antibodies revealed a specific interaction between C16orf74 and PPP3CA. Endogenous PPP3CA interacted with the phosphorylated form of endogenous C16orf74 (arrow). **C.** Interactions of wild-type C16orf74 (WT) and mutants of C16orf74 with PPP3CA, as assessed by IP analysis. Expression vectors for myc-His-tagged PPP3CA and Flag-tagged C16orf74 constructs were doubly transfected into HEK293T cells. C16orf74 (anti-Flag) was IP, and the indicated molecules were immunoblotted (IB) in western blot analysis. WT, replacement (T44A; non-phosphorylated form of C16orf74) and deletion mutants (∆PDIIIT; deletion mutant of PPP3CA binding motif) were analyzed. PPP3CA bound to wild-type C16orf74 but not the non-phosphorylated form of C16orf74 or the deletion mutant of the PPP3CA binding motif. **D.** Subcellular localization of C16orf74 (wild type or ∆PDIIIT) and PPP3CA in mammalian cells. Flag-tagged (green) C16orf74 (wild type or ∆PDIIIT) and myc-tagged (red) PPP3CA constructs were cotransfected into COS-7 cells and subjected to immunocytochemical staining. Flag-C16orf74 (wild type) and myc-PPP3CA colocalized on the under the cytoplasmic membrane of COS-7 cells (yellow), but Flag-C16orf74 (∆PDIIIT) did not colocalize with myc-PPP3CA, which was present diffusely in the cytoplasm. **E.** Interactions of endogenous C16orf74 with PPP3CA as assessed by IP analysis. The phosphorylated form (arrow) of endogenous C16orf74 in KLM-1 cells, as examined by western blot analysis using an anti-C16orf74 polyclonal antibody. Pre IP (left; non-immunoprecipitated by PPP3CA), the phosphorylated form of C16orf74 (upper band) disappeared when the cell lysate was incubated with lambda phosphatase (PPase (+)). Immunoprecipitation by PPP3CA (right) revealed that the phosphorylated form of C16orf74 (upper band) interacted with PPP3CA, whereas the non- phosphorylated form of C16orf74 did not. **F.** Invasion activity of wild-type C16orf74 (WT) and the two mutants (T44A: non-phosphorylated form of C16orf74; and ∆PDIIIT, deletion mutant of the PPP3CA binding motif). The WT-C16orf74 expression vector, T44A-C16orf74 expression vector, ∆PDIIIT-C16orf74 expression vector, and Mock vector were each transfected into NIH3T3 cells. The Matrigel invasion assay revealed an enhanced cell number for WT-C16orf74-over-expressing cells (3.4-fold, *P = 0.013) but not so enhanced for ∆PDIIIT-C16orf74-over-expressing cells (1.4-fold, **P = 0.017) or T44A-C16orf74-over-expressing cells (2.3-fold,***P = 0.038).

Because the biological functions of C16orf74 are unknown, we searched for proteins that interact with C16orf74 using the TAP (tandem affinity purification) system combined with mass-spectrometry analyses (see Materials and Methods). We identified the several candidates of C16orf74-binding proteins. Among them, the protein phosphatase 3 catalytic subunit alpha (PPP3CA), an isoform of Calcineurin A subunit (CnA) which is a serine-threonine calcium/calmodulin-dependent phosphatase, as a candidate C16orf74-interacting protein (Supplemental Figure 4). We focused on PPP3CA as a C16orf74-binding partner because C16orf74 protein contains a PPP3CA consensus binding sequence (PXIXIT motif) by *in silico* analysis. The exogenous interaction of the proteins was confirmed by an immunoprecipitation (IP) assay using anti-myc and anti-Flag antibodies (Figure 5A), and their endogenous interaction in PK-1 cells was also confirmed by IP assay using anti-C16orf74 and anti-PPP3CA antibodies (Figure 5B). Notably, PPP3CA bound preferentially to the phosphorylated form of C16orf74 (Figure 5A, arrow). We subsequently determined whether T44 phosphorylation of C16orf74 is required for the interaction with PPP3CA. Substitution of T44 to alanine (T44A) almost completely abolished the interaction with PPP3CA (Figure 5C), indicating the significance of T44 phosphorylation for interaction of these two proteins. Moreover, since C16orf74 possesses a PDIIIT sequence (Supplemental Figure 2), a PPP3CA-binding motif (PxIxIT) conserved in nuclear factor of activated T cells (NFATs) transcriptional factor and other PPP3CA-binding partners [16–18], we investigated the importance of this motif sequence for the C16orf74-PPP3CA interaction. IP assays demonstrated that a mutant in which the PDIIIT sequence within C16orf74 is deleted (∆PDIIIT) completely abolished the interaction with PPP3CA (Figure 5C), suggesting that C16orf74 binds to PPP3CA *via* PDIIIT, the PxIxIT binding motif, although we can not exclude a possibility that the conformational change of protein lacking the domain simply diminished the interaction.

**Table 2:** Univariate and multivariate analysis of prognostic factors using the Cox proportional hazards model

Factor		Univariate analysis			Multivariate analysis		
		RR	95% CI	*p*-value	RR	95% CI	*p*-value
Age (≤65 / 66<)		1.01	0.63-1.62	0.95			
Sex (male / female)		0.85	0.52-1/38	0.51			
Tumor size (≤30 / 30<)		1.33	0.82-2.15	0.25			
Tumor site (Ph / Pb, Pt)		0.71	0.41-1.24	0.23			
Lymph node metastasis (- / +)		2.24	1.31-3.81	0.003*	2.61	1.51-4.53	0.0006*
Lymphatic invasion (- / +)		1.42	0.82-2.45	0.21			
Venous invasion (- / +)		2.11	0.95-4.67	0.066			
Perineural invasion (- / +)		2.18	0.87-5.48	0.096			
C16orf74 expression (low / high)		1.71	1.06-2.77	0.028*	2.05	1.25-3.36	0.004*

RR: relative risk, CI: confidence interval, Ph: pancreatic head, Pb: pancreatic body, Pt: pancreatic tail, *p < 0.05.

To investigate the subcellular localization of C16orf74 and PPP3CA in mammalian cells, we cotransfected Flag-tagged C16orf74 (wild type or ∆PDIIIT) and myc-tagged PPP3CA constructs into COS-7 cells and performed immunocytochemical staining analysis. Flag-C16orf74 (wild type: WT) and myc-PPP3CA colocalized on the same side of cytoplasmic membrane of COS-7 cells. By contrast, Flag-C16orf74 (∆PDIIIT) did not colocalize with myc-PPP3CA and was diffusely located in the cytoplasm of COS-7 cells. Furthermore, we confirmed that the non-phosphorylated form of C16orf74 (the T44A substitutant) did not interact with PPP3CA in the IP assay (Figure 5C), and phosphatase treatment interrupted the C16orf74-PPP3CA interaction (Figure 5E).

Because PPP3CA is reported to play an important role in invasiveness in cancers [19–21], we examined whether the C16orf74-PPP3CA interaction is required for invasion activity. We performed a Matrigel invasion assay using substitutants of C16orf74 that are unable to interact with PPP3CA (T44A and ∆PDIIIT). A decrease in invasion activity was observed for T44A or ∆PDIIIT compared with the wild type (Figure 5F). These findings support the hypothesis that the C16orf74-PPP3CA interaction plays a critical role in the invasiveness of PDAC cells.

## DISCUSSION

In this study, we have focused on *C16orf74* (NM_206967.2), which is frequently over-expressed in PDACs but exhibits a restricted expression pattern in normal adult tissues. *C16orf74* is a functionally unknown gene and was identified as *Homo sapiens* chromosome 16 open reading frame 74. *C16orf74* has been reported as part of gene expression profiles such as the inflammation-associated gene profile [9] and several cancer prognosis-associated gene profiles [10, 12–15]. mRNA expression of *C16orf74* has been reported as a factor for good prognosis in bladder cancer but controversially it was suggested to be associated with lymph node metastasis in tongue cancer [12, 15].

In this report, we elucidated the detailed structure of three transcriptional variants of *C16orf74*. The V1 variant was specifically upregulated in PDAC cells, and its gene product plays a crucial role in pancreatic cancer proliferation and invasion. Immunohistochemical staining analysis of the PDAC tissue microarray revealed that more than half of PDAC tumors (46/81, 56.8%) had high expression of C16orf74 proteins. Multivariate Cox proportional hazards analysis demonstrated that C16orf74 expression level is an independent prognostic factor for postoperative survival of PDAC patients. Hence, our data suggest that C16orf74 has an oncogenic function, is a biomarker for poor prognosis, and could be a promising therapeutic target for pancreatic cancer. Our results are in contrast to those for bladder cancer, for which *C16orf74* is a good prognostic factor [12]. This discrepancy might be due to differences of mRNA expression pattern of each variant of *C16orf74* in different cancer type, although further analysis will be necessary. We also searched the somatic mutations and copy number alterations in *C16orf74* gene in PDACs by COSMIC database (Catalogue of somatic mutations in cancer: http://cancer.sanger.ac.uk/cosmic), but found no somatic mutations and copy number alterations in this genes.

In this report, we demonstrated for first time that the C16orf74-PPP3CA interaction *via* PDIIIT, the PPP3CA-binding motif (PxIxIT) and T44 phosphorylation is potentially indispensable for PDAC invasiveness. PPP3CA is reported to interact with NFATs (NFATc1, NFATc2, NFATc3 and NFATc4) transcriptional factors, and have a crucial role in the regulation of immune response [26], and invasiveness of cancer cells [19–21, 27, 28]. It was reported that down-regulation of PPP3CA gene expression could decrease the proliferation, the cell migration and invasion of small-cell lung cancer. In addition, *in vivo* down- or up-regulation of PPP3CA gene expression could decrease or increase the bone metastasis [29]. The NFATs are regulated at the level of their subcellular-localization *via* the action of the Ca^2+^/calmodulin-dependent serine/threonine phosphatase calcineurin (Cn) [30]. The increase of intracellular Ca^2+^ level leads to activation of Cn, thereby induces nuclear-translocation of transcriptional factor NFATs [31, 32]. More importantly, PPP3CA-NFATs pathway is reported to be crucial in pancreatic cancer growth and invasion [33, 34]. We demonstrated here that the inhibition of the interaction between C16orf74 and PPP3CA using T44A and ∆PDIIIT constructs led to the suppression of the invasiveness of cancer cells. Accordingly, these evidences suggest that the up-regulation of the PPP3CA pathway *via* the C16orf74-PPP3CA interaction under the plasma membrane of PDAC cells should lead to constitutive nuclear-translocation and transcriptional activation of NFATs, resulting in up-regulation of cancer-related genes such as c-myc leading to enhancement of cell proliferation and invasiveness, although further elucidation of mechanism of NFATs signaling activation in C16orf74-overexpressing PDAC cells will be necessary. Moreover, since *C16orf74* is reported to be a gene associated with hypoxia [35, 36], we examined the involvement of *C16orf74* expression in Hypoxia by using public genome-wide gene expression profiling database, and found that *C16orf74* gene is a Hypoxia- inducible factor 1(HIF1)-regulated gene [37]. Further elucidation of biological roles of *C16orf74* as a HIF-1 regulated gene in PDAC cells will be necessary.

In conclusion, our findings clearly demonstrate that C16orf74 is overexpressed in the vast of majority of human PDAC, and likely plays a significant role in pancreatic pancreatic cell growth and invasion through its interaction with PPP3CA. We suggest that C16orf74 could be a useful prognostic marker for PDAC and any means to inhibit C16orf74-PPP3CA complex formation could be promising to treat PDAC.

## MATERIALS AND METHODS

### Cell lines and clinical materials

Cell lines: The PDAC cell lines Capan-1, Capan-2, MIA-Paca2, Panc-1, and Aspc-1, the embryonic kidney cell line HEK293, and NIH3T3 were purchased from American Type Culture Collection (ATCC, Rockville, Maryland). The PDAC cell lines PK-59 and KLM-1 were provided by the Cell Resource Center for Biomedical Research, Tohoku University (Sendai, Japan). All cells were cultured in appropriate media: RPMI-1640 (Sigma, St. Louis, MO) for Capan-1, Capan-2, Aspc-1, KLM-1, and PK-59 and Dulbecco's modified Eagle's medium (Invitrogen, Carlsbad, CA) for MIApaca-2, Panc-1, and NIH3T3. Each medium was supplemented with 10% fetal bovine serum (Cansera) and 1% antibiotic/antimycotic solution (Sigma). Cells were maintained at 37°C in an atmosphere of humidified air with 5% CO_2_. Clinical samples (pancreatic cancer and normal pancreatic duct) were obtained from surgical specimens that were resected at Hokkaido University Hospital. Informed consent was obtained from patients, and the study was approved by the institutional review board of Hokkaido University Hospital (ID; 014-0224). Normal tissue sections were purchased from Biochain (Hayward, CA, USA).

### Northern blot analysis

Human multiple-tissue Northern blots (Clontech, Palo Alto, CA) or a membrane including RNA samples from cancer cell lines and normal organs (Clontech) were hybridized with a [^32^P]-dCTP-labeled amplification product of *C16orf74* prepared by RT-PCR (see below). Pre-hybridization, hybridization and washing were performed according to the supplier's recommendations. The blots were autoradiographed with intensifying screens at -80°C for 10 days. A specific probe for V1 and V2 was prepared using exon 1 and 2 of *C16orf74*, and the V1-3 common probe was prepared using exon 4 as the template.

### cDNA library screening

We constructed a cDNA library using a Superscript^TM^ plasmid system with Gateway^TM^ technology for cDNA synthesis, a cloning kit (Invitrogen) and poly(A)+ RNA obtained from pancreatic cancer cell line Capan-1. We screened 3×10^6^ independent clones of this library using the same cDNA probes used for Northern blot analysis.

### Semi-quantitative RT-PCR analysis

The extraction of total RNAs from clinical samples of laser-microdissected cells was performed as described previously [8]. The amplified RNA or total RNA was reverse transcribed to generate single-stranded cDNAs using a random primer (Roche) or oligo(dT)_16_ primer with Superscript II reverse transcriptase (Roche). We prepared appropriate dilutions of each single-stranded cDNA for subsequent PCR amplification by monitoring tubulin, alpha 3 *(TUBA3)* as a quantitative control. The primer sequences were 5’-CTTGGGTCTGTAACAAAGCATTC-3’ and 5’-AAGGATTATGAGGAGGTTGGTGT-3’ for *TUBA3* and 5’-GTCCTGAAAGTCAAGCACCTG-3’ and 5’-GAAGTTCTTGTTGGTGCTTATGG-3’ for C16orf74. All reactions involved initial denaturation at 94°C for 2 min, followed by 22 cycles (for *TUBA3*) or 28 cycles (for *C16orf74*) of 94°C for 30 s, 58°C for 30 s, and 72°C for 1 min on a GeneAmp PCR system 9700 (PE Applied Biosystems).

### Preparation of recombinant C16orf74 (rC16orf74)

The full-length *C16orf74*-V1 cDNA fragment was generated by RT-PCR and cloned into pGEX-6P-1 (Life Technologies). Recombinant GST-tagged protein was expressed in *Escherichia coli* BL21 codonplus (Agilent). After induction with 0.2 mM isopropyl-β-D-thiogalactopyranoside (IPTG) at 25°C, the bacterial pellet was suspended in lysis buffer and purified with Glutathione Sepharose resin (GE Healthcare) in accordance with the manufacturer's instructions. The GST-C16orf74 fusion protein was digested with PreScission Protease for 20 h and then further purified by column chromatography using a Mono Q HR5/5 column (GE Healthcare) and an ÄKTA purifier (GE Healthcare).

### Polyclonal antibody against C16orf74

Polyclonal anti-C16orf74 antibodies were raised in rabbits against purified C16orf74 recombinant protein (rC16orf74) (Medical and Biological Laboratories, Nagoya, Japan). The high-titer antiserum was affinity-purified using rC16orf74 and a C16orf74-peptide (HDEAPVLNDKHLDVPDI) coupled support (Affigel 15; BioRad, Hercules, CA, USA). This antibody can recognize V1 and V3 of C16orf74 protein, but not V2.

### Western blot analysis

The cultured cells were washed twice with PBS and harvested in lysis buffer (150 mM NaCl, 1% Triton X-100, 50 mM Tris-HCl pH 7.4, 1 mM DTT, and 1X complete Protease Inhibitor Cocktail (Boehringer)). After the cells were homogenized and centrifuged at 10,000x*g* for 30 min, the protein concentration of the supernatant was standardized by the Bradford assay (Bio-Rad). Proteins were separated by 12% SDS-PAGE and immunoblotted with antibody. The antibodies used in the western blot analysis were rat anti-HA, mouse anti-HA (sc-7392, Santa Cruz), anti-Flag-M2, rabbit anti-Flag (F7425, SIGMA), mouse anti-beta-actin (A5441, SIGMA), mouse anti-PPP3CA (sc-17808, Santa Cruz), and rabbit anti- C16orf74 antibodies.

### Lambda-phosphatase assay

The lysate of KLM-1 cells was treated with lambda protein phosphatase (New England BioLabs) according to the manufacturer's instructions. In the PPP3CA interaction assay, the KLM-1 cell lysate was incubated with lambda protein phosphatase, immunoprecipitated by mouse monoclonal PPP3CA (sc-17808, Santa Cruz) and immunoblotted with rabbit polyclonal C16orf74, as in the western blot analysis.

### Immunochemical staining

Cultured cells were fixed with PBS containing 4% paraformaldehyde for 20 min at 4°C and permeabilized with PBS containing 0.1% Triton X-100 for 2.5 min at room temperature. The cells were blocked with 3% BSA in PBS for 1 hr and then incubated with rabbit anti-C16orf74 or mouse anti-Flag-M2 antibody for 1 hr at room temperature, followed by incubation with Alexa488-conjugated secondary antibody (Molecular Probes). Nuclei were counter-stained with DAPI. Fluorescent images were obtained by confocal microscopy (Leica).

Paraffin-embedded sections of pancreatic cancer and normal tissues were treated with xylene, and antigen retrieval was performed by microwaving in antigen-retrieval buffer (DAKO). Endogenous peroxidase activity was blocked by incubation with Peroxidase Blocking Reagent (DAKO). Sections were blocked with Protein Block Serum-Free (DAKO) for 30 min and then incubated with anti-C16orf74 antibody for 30 min at room temperature. After washing with PBS, the sections were incubated with HRP-conjugated anti-rabbit IgG (DAKO) and color developed with DAB. Finally, the sections were counterstained with hematoxylin. Images were obtained by a CCD camera attached to a microscope (Olympus).

### Tissue microarray

The tissue microarray (TMA) was constructed as described previously [38]. PDAC tissue samples were obtained from 99 PDACs resected at Hokkaido University Hospital between 1999 and 2005. C16orf74-positive was defined as stronger cytoplasmic staining of C16orf74 in pancreatic cancer cells than in normal pancreas tissue. Immunoreactivity was evaluated on a scale considering the extent (score- for positive cancer cells ≤10% or score+ for positive cancer cells > 10%) of staining. We classified the TMA cases into a low-expression group and high-expression group, as defined by an extent score of - or +, respectively, if defined as such by three reviewers (T.N., S.S., T.K.). Eighteen patients without information about survival and clinicopathological factors were omitted from the analysis, and 81 patients were finally subjected to the analysis (follow-up term from 2.0 to 156.3 months). Informed consent was obtained from patients, and the study was approved by the Institutional Review Board of Hokkaido University Hospital (No. 014-0221). Patient tumor stage was classified according to the tumor-node-metastasis (TNM) classification of the Union for International Cancer Control (UICC) [39].

### Gene silencing of C16orf74 in PDAC Cells

To knockdown the gene expression of *C16orf74* in PDAC cells, we used a psiU6BX3.0 vector to express a short-hairpin RNA against the target gene, as described previously [40]. The target sequences for C16orf74 were 5V- CGACAAGCACCTGGACGTG-3V(C16orf74-si1), 5V- ATGTGTGTCAGCAGCAGCA-3V (C16orf74-si2), 5V- GTTGTTTTACAGATACGGA-3V (C16orf74-si3), and 5V-GAAGCAGCACGACTTCTTC-3V (for siEGFP as a negative control). The human PDAC cell lines KLM-1 (5×10^5^ cells per dish) and PK-59 (2×10^5^ cells per dish) were plated on 10-cm dishes and transfected with psiU6BX vectors including the target sequences for *EGFP* and *C16orf74* using Nucleofector (Amaxa Biosystems, Cologne, Germany) according to the manufacturer's protocol. Cells were selected in medium containing 500 µg/mL G418 for 7 days and then harvested to analyze the effect of knockdown on *C16orf74* expression. The primers for these RT-PCR experiments were the same as those described above. For the colony formation assay, transfectants expressing siRNA were grown for 7 days in media containing G418. After fixation in 4% paraformaldehyde, the transfected cells were stained with Giemsa solution to assess colony formation. Cell viability was quantified using cell counting kit-8 (Dojindo, Kumamoto, Japan).

### Matrigel invasion assay

NIH3T3 cells transfected with the pCAGGS-C16orf74-Flag vector or empty pCAGGS/ Flag mock vector were grown to near confluence in culture medium containing 10% FBS. The cells were harvested by trypsinization, washed, and finally suspended in serum-free culture medium at a concentration of 2 × 10^5^ cells/mL. Before preparing the cell suspension, the dried layer of the Matrigel matrix (Becton Dickinson Labware) was rehydrated with culture medium for 2 hr at room temperature. Culture medium (0.75 mL) containing 10% FBS was added to each lower chamber in 24-well Matrigel invasion chambers, and 0.5 mL (1 × 10^5^ cells) of cell suspension was added to each insert of the upper chamber. The inserts were incubated for 22 hr at 37°C. After incubation, the chambers were processed; cells invading through the Matrigel were fixed and stained by Giemsa as directed by the supplier (Becton Dickinson Labware). An identical experiment was performed for NIH3T3 cells transfected with the pCAGGS-ΔPDIIIT C16orf74-Flag vector or pCAGGS- T44A mutant C16orf74-Flag vector.

### Cell proliferation assay

HEK293T cells were plated into a 6-well microtiter plate and transiently transfected with pCDNA3.1 Myc-His-*C16orf74* or pcDNA3.1 Myc-His mock control expression vector using Fugene 6 transfection reagent (Roche) according to the manufacturer's instructions. Seven days after transfection, cell viability was quantified using cell counting kit-8 (Dojindo).

### Immunoprecipitation and mass spectrometric analysis of C16orf74-associated complexes

To isolate proteins that associated directly with C16orf74, we used the TAP system (tandem affinity purification system) combined with mass spectrometry analysis. The TAP procedure was performed essentially as described previously with some modifications [41]. The TAP vector expresses an amino-terminal HA-tagged target protein with carboxy-terminal TAP tags in mammalian cells under the control of a cytomegalovirus promoter. The TAP vector with HA-tagged C16orf74 or Mock-HA was transiently transfected into Panc-1 cells, followed by lysis in lysis buffer [50 mM Tris-HCl (pH 8.0), 150 mM NaCl, 0.5% NP40, Protease Inhibitor Cocktail Set III (Calbiochem, San Diego, CA)]. Equal amounts of total proteins were incubated at 4°C for 1 hr with anti-HA antibody. Immunocomplexes were incubated with protein G-Sepharose (Zymed Laboratories, South San Francisco, CA) for 1 hr and washed with lysis buffer. Coprecipitated proteins were separated in a 5% to 20% gradient SDS-PAGE and stained by silver-staining kits (Wako, Osaka, Japan). Bands that differentiated proteins precipitated with HA-C16orf74 from those precipitated with HA-Mock were excised, digested in gel with trypsin, and analyzed for peptide-mass fingerprints using an AXIMA-CFR +6 mass spectrometer (Shimadzu Corporation, Tsukuba, Japan). Peptide masses were searched with 10-ppm mass accuracy, and protein database searches were performed using the database-fitting program IntelliMarque (Shimadz).

### Immunoprecipitation and western blotting

Transfected cells were harvested with RIPA buffer (150 mM NaCl, 1% NP-40, 50 mM Tris-HCl (pH 8.0), 0.1% SDS, 0.5% sodium deoxycholate, and 1X protease inhibitor cocktail set III (Calbiochem)) 2 days after transfection. Immunoprecipitation was performed with mouse anti-Myc (9E10, Santa Cruz) antibody or anti-Flag-M2 (F3165, SIGMA) antibody. The antibodies were removed by incubation with protein G Sepharose (Zymed/Invitrogen), and the wash step was repeated 5 times. Proteins were extracted with SDS sample buffer and separated by 10-20% gradient SDS-page (Bio-Rad). To examine the interaction of Flag-C16orf74 with Myc-PPP3CA, we analyzed the immune complexes by western blotting with rabbit anti-Flag and anti-Myc antibodies. To examine the interaction of endogenous C16orf74 with endogenous PPP3CA, Capan-1 cells were harvested in RIPA buffer, and immunoprecipitation was performed with mouse anti-PPP3CA (sc-17808, Santa Cruz) antibody or rabbit anti-C16orf74 (immunized with full-length recombinant C16orf74 protein) antibodies. Then, we collected the antibodies with protein G Sepharose (Zymed/Invitrogen) and repeated the wash steps 5 times. Proteins were extracted with SDS sample buffer and separated by 10-20% gradient SDS-PAGE (Bio-Rad). We analyzed the immune complexes by Western blotting with mouse anti-PPP3CA (sc-17808, Santa Cruz) antibody, or rabbit anti-C16orf74 antibodies.

### Construction of the expression vector

The entire coding sequence of C16orf74 V1 cDNA was amplified by RT-PCR with the primers C16orf74 V1-forward (5’-CCGGAATTCGACATGGGGCTTAAGATGTCC-3’) and C16orf74 V1-reverse (5’-CCGCTCGAGGGCTTCTGGGTCGATTTCTCC-3’). The product was inserted into the *Eco*RI and *XhoI* sites of pCAGGS to express a Flag-tagged protein [42]. The entire coding sequence of PPP3CA cDNA was amplified by RT-PCR with the primers 5’-CGCGGATCCATGTCCGAGCCCAAGGCAATT-3’ (forward) and 5’-CCGCTCGAGCTGAATATTGCTGCTATTACT-3’ (reverse) and inserted into the *BamHI* and *XhoI* sites of pcDNA3.1(+)/Myc-His A (Invitrogen) to express a Myc-tagged protein.

### Statistical analysis

The results are presented as the mean ± SEM of at least 3 independent experiments. The data were analyzed using the Student's *t*-test, Mann-Whitney U test, Fisher's exact test, or χ2 test as appropriate. Overall survival was calculated from the date of operation to the date of last follow-up or date of patient death. Univariate survival analysis was performed according to the Kaplan-Meier method, and survival differences were estimated by the log-rank test. Multivariate analysis was conducted using the Cox proportional hazards regression model. A *p*-value < 0.05 was considered significant, and the confidence interval (CI) was determined at the 95% level. All data were analyzed using JMP^®^ 10 software.

## SUPPLEMENTARY MATERIALS FIGURES AND TABLES


