# Supplementary material for: Overexpression of C16orf74 is involved in aggressive pancreatic cancers
Source: Oncotarget. 2016 Jul 28;8(31):50460–75. doi: 10.18632/oncotarget.10912 (PMC5584151; doi:10.18632/oncotarget.10912)
Supplement: Supplementary file 1 [file oncotarget-08-50460-s001.docx]

Overexpression of C16orf74 is involved in aggressive pancreatic cancers

**Supplementary Material**


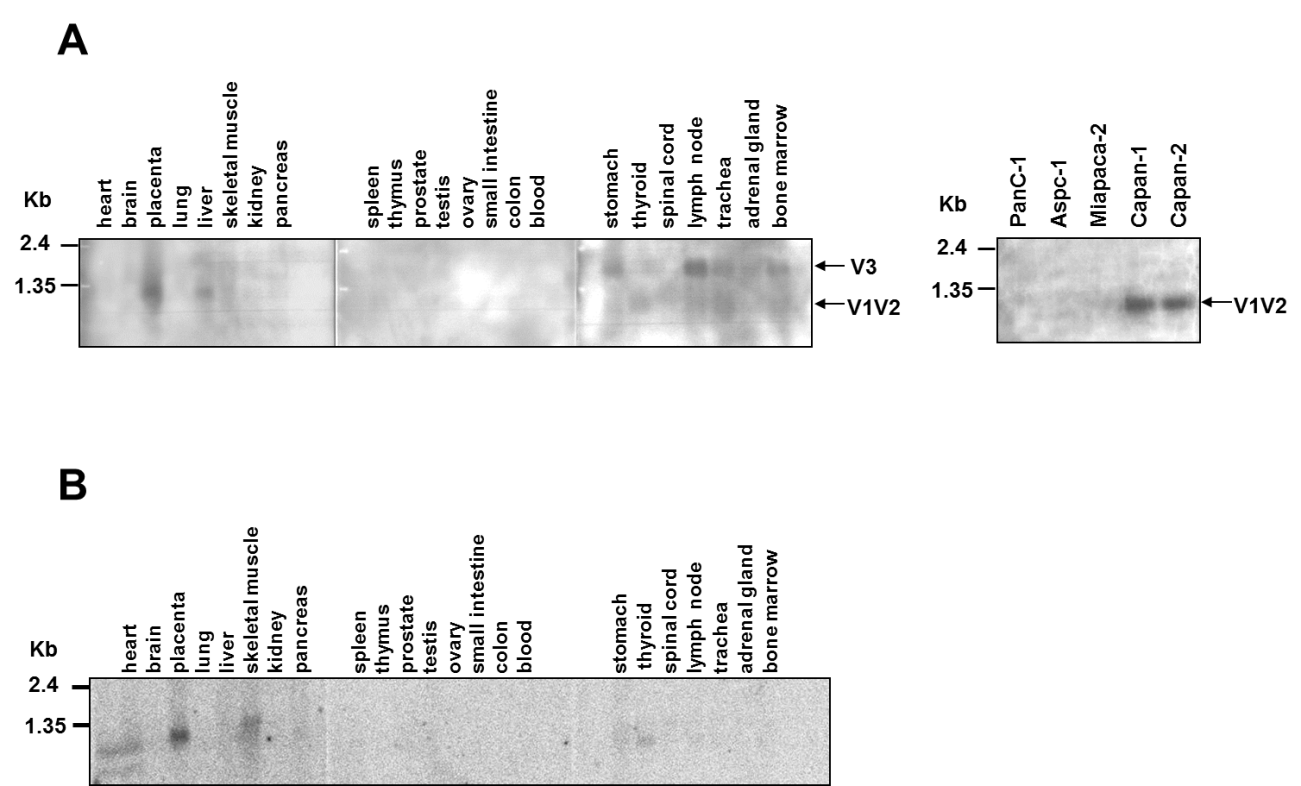


Supplemental Figure 1.

(A) Northern blot analysis of 23 normal human tissues (left) and 5 pancreatic cancer cell lines (right) using the common probe indicated that V1/V2 variants are specifically expressed in cancer cells.

(B) Northern blot analysis of 23 normal human tissues using the V1/V2-specific probe.


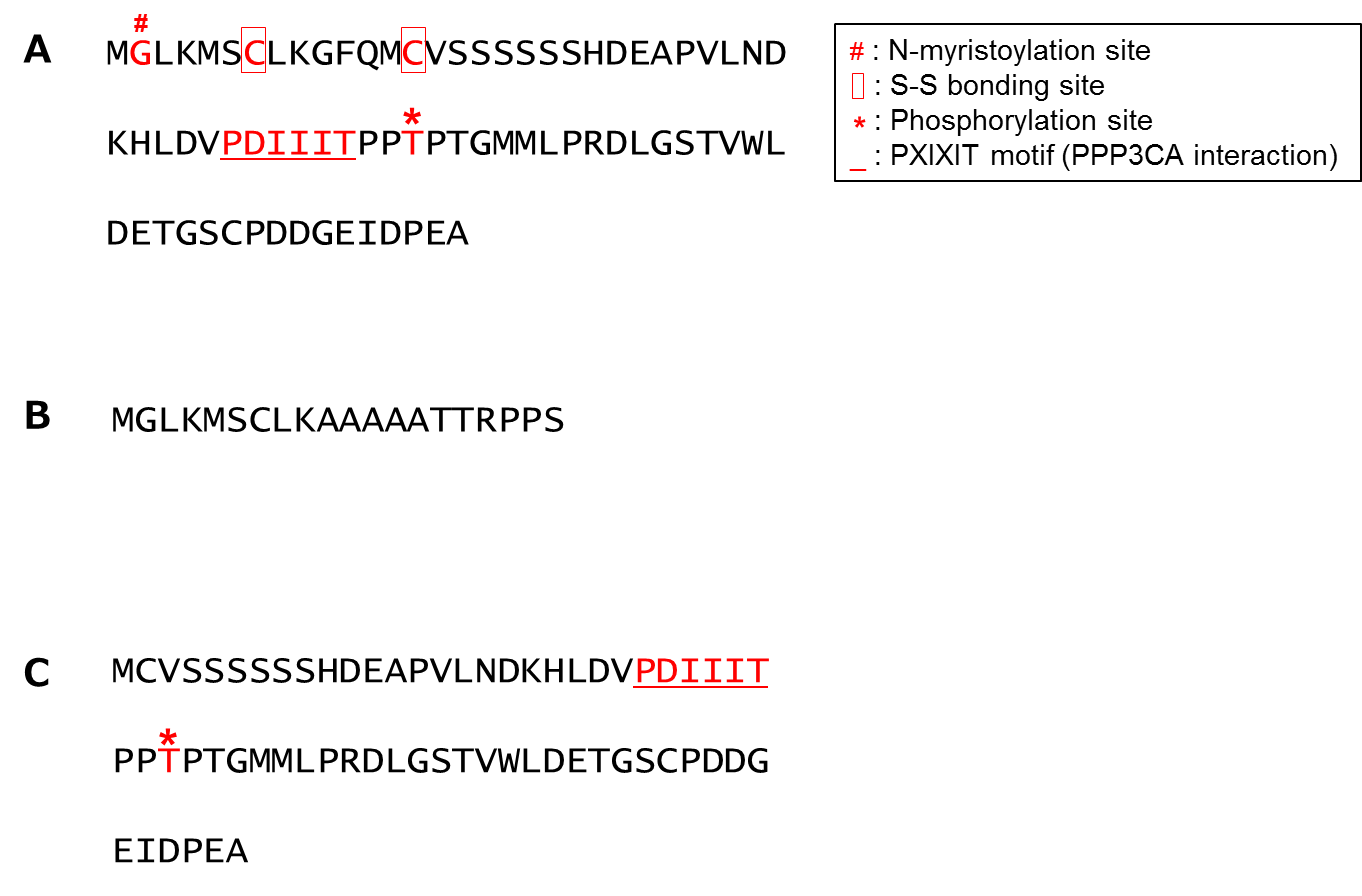


Supplemental Figure 2.

Deduced amino acid sequence of C16orf74. The predicted sites of protein modification and interaction are indicated (A:V1 variant, B:V2 variant, C:V3 variant).

A


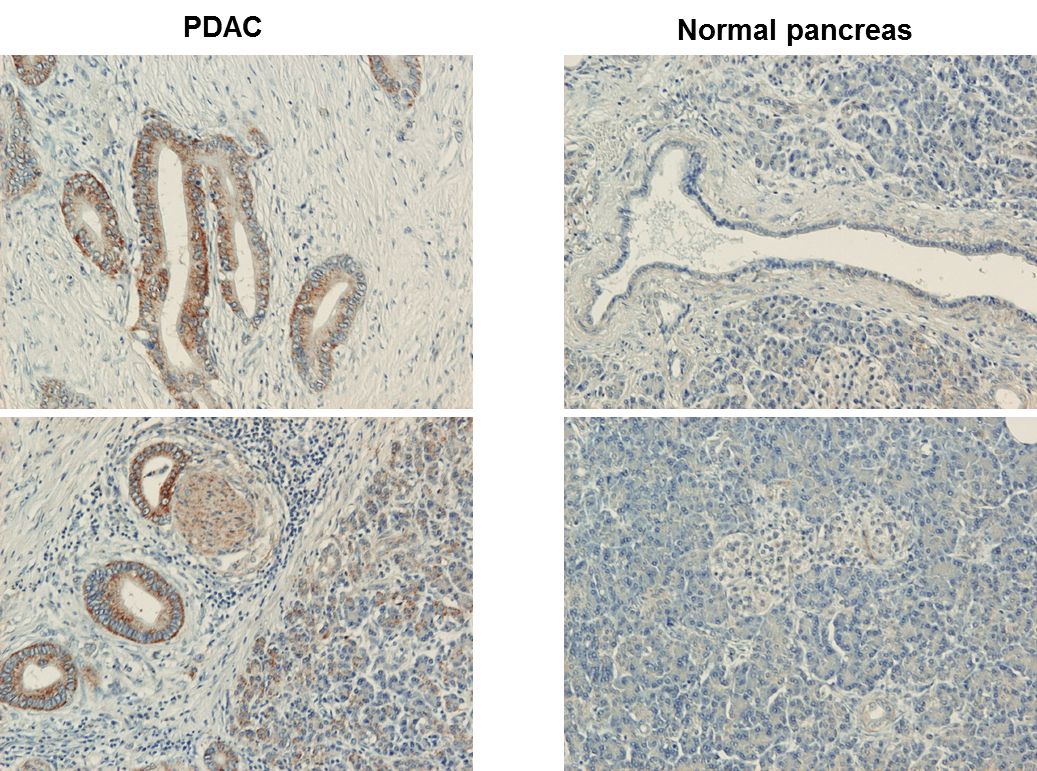


Supplemental Figure 3.

(A) Immunohistochemical analysis of pancreatic ductal adenocarcinoma (PADC) and normal pancreas sections revealing enhanced expression of C16orf74 in the cancer cells.

B


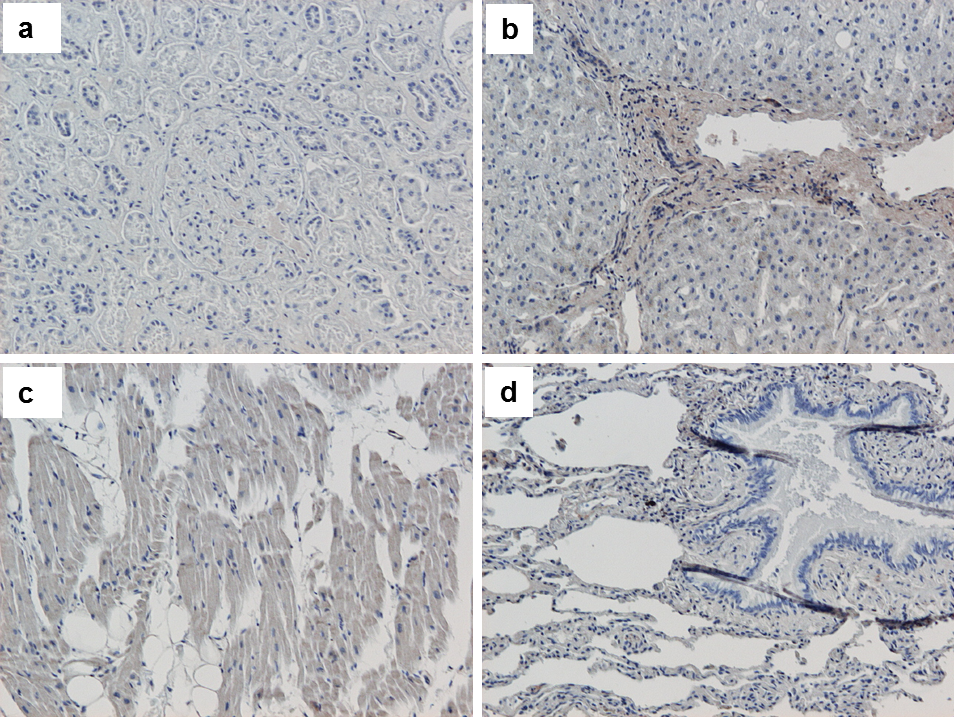


Supplemental Figure 3.

(B) Immunohistochemical analysis of normal vital organ tissue sections revealing no expression of C16orf74 in the kidney (a), liver (b), heart (c), or lung (d).


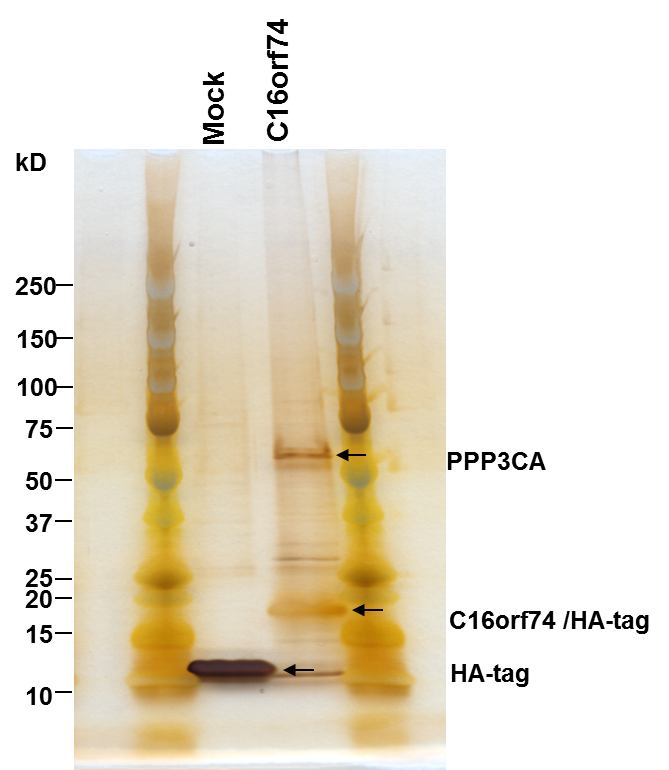


Supplemental Figure 4.

Proteins coprecipitated using the TAP system (tandem affinity purification system) were separated by 5% to 20% gradient SDS-PAGE and stained by silver staining. Bands that differentiated proteins precipitated with HA-C16orf74 from those precipitated with HA-Mock were excised, and Mass spectrometry analyses identified calcineurin alpha subunit phosphatase (PPP3CA) as a candidate C16orf74-interacting protein.
